# Objectively Assessed Daily Steps—Not Light Intensity Physical Activity, Moderate-to-Vigorous Physical Activity and Sedentary Time—Is Associated With Cardiorespiratory Fitness in Patients With Schizophrenia

**DOI:** 10.3389/fpsyt.2019.00082

**Published:** 2019-02-26

**Authors:** John A. Engh, Jens Egeland, Ole A. Andreassen, Gry Bang-Kittilsen, Therese T. Bigseth, Tom L. Holmen, Egil W. Martinsen, Jon Mordal, Eivind Andersen

**Affiliations:** ^1^Division of Mental Health and Addiction, Vestfold Hospital Trust, Tønsberg, Norway; ^2^Department of Psychology, University of Oslo, Oslo, Norway; ^3^NORMENT, KG Jebsen Centre for Psychosis Research, Oslo, Norway; ^4^Division of Mental Health and Addiction, Institute of Clinical Medicine, University of Oslo, Oslo, Norway; ^5^Faculty of Humanities, Sports and Educational Science, University of South-Eastern Norway, Horten, Norway

**Keywords:** schizophrenia, lifestyle, cardiovascular disease, daily steps, physical activity, cardiorespiratory fitness, accelerometer, sedentary time

## Abstract

People with schizophrenia often have an unhealthy sedentary lifestyle with low level of physical activity and poor cardiorespiratory fitness—an important predictor of cardiovascular disease. We investigated the relations between cardiorespiratory fitness and both sedentary time and different aspects of physical activity, such as daily steps, light intensity physical activity, and moderate-to-vigorous physical activity. Using accelerometer as an objective measure of sedentary time and physical activity we estimated their relations to cardiorespiratory fitness in 62 patients with schizophrenia with roughly equal gender distribution, mean age of 36 and 15 years illness duration. We found a significant association between daily steps and cardiorespiratory fitness when accounting for gender, age, sedentary time, light intensity physical activity, and respiratory exchange ratio (maximal effort). Moderate-to-vigorous physical activity was not significantly associated with cardiorespiratory fitness. In conclusion, the amount of steps throughout the day contributes to cardiorespiratory fitness in people with schizophrenia, independently of light intensity physical activity and sedentary time. We did not find a significant relationship between moderate-to-vigorous physical activity and cardiorespiratory fitness. This may have implications for the choice of strategies when helping patients with schizophrenia improve their cardiorespiratory fitness.

## Introduction

People with schizophrenia are prone to overweight, diabetes and chronic metabolic disease (1) and often have a deleterious lifestyle, including low levels of moderate-to-vigorous physical activity (MVPA) and high levels of sedentary time (2). The average life expectancy is 15–20 years shorter than the general population, and cardiovascular disease (CVD) is the largest contributing factor to the increase in mortality (3). Low cardiorespiratory fitness (CRF) is a strong independent risk factor for all-cause mortality (4, 5) associated with low life expectancy and increased risk for CVD in the general population (4–6) and in schizophrenia (7, 8). Thus, knowledge on how to effectively improve CRF in schizophrenia is urgently needed. The beneficial effects of MVPA and the smaller effects of light intensity physical activity (PA) on CRF in the general population are well documented (9, 10). Less is known about the relations in people with schizophrenia. Another aspect of interest is the extent sedentary time influences CRF in the patient group. Sedentary time refers to the time spent for any duration or in any context in sedentary behaviors, defined as any waking behavior characterized by an energy expenditure equal to or less than 1.5 the resting metabolic rate while in a sitting, reclining or lying posture (11–13). Recent studies suggest that sedentary time is an important determinant of CRF levels, which is independent of physical activity (14, 15) A third aspect of interest is the total PA level. The number of daily steps have been associated with positive health effects in the general population (16) and in individuals with high cardiovascular risk (17). Studies on healthy overweight persons have indicated that reduced total daily PA causes reduction in CRF as well as worsening of other cardiometabolic health outcomes (18, 19). Increasing the total PA by walking (i.e., daily steps), as well as breaking the sedentary habit could be a path to improved CRF distinct from light intensity PA and MVPA (20, 21). It is not known whether the potential health effects facilitated by daily steps in people with schizophrenia are cumulative, increasing with the amount of total PA. The aim of the current study was to examine whether objectively assessed light intensity PA, MVPA, total daily steps, and sedentary time, exert *independent* influences on CRF in people with schizophrenia.

## Materials and Methods

### Participants

Sixty-two patients were recruited from the main study Effects of physical activity in psychosis (EPHAPS) from August 2014 through September 2016 in catchment area-based and publicly funded outpatient psychiatric clinics in Vestfold County, Norway. A subgroup of the patients was referred from primary health care to the outpatient clinics for specific participation in the project. Patients diagnosed with schizophrenia spectrum disorder established using SCID I (22) who were aged 18–67 and understood and spoke a Scandinavian language were eligible for the study. Interviews were conducted by a clinical psychologist or a specialist in psychiatry. For further details on study design see Engh et al. (23) and on patient recruitment, study eligibility and data collection see Engh et al. (23) and Andersen et al. (24). All except one patient received antipsychotic treatment. Demographic and clinical characteristics are presented in Table 1.

**Table 1:** Demographic and clinical characteristics, physical activity and sedentary time of the participants.

	***N***	**Value**	***SD***
Age (years), mean	62	36.3	13.7
Gender (women, %)	62	27 (43.5)	–
Duration illness (years)	61	14.9	12.2
Smokers (%)	62	40 (64.5)	–
Body mass index (kg*m^−2^)	62	29.2	5.7
PANSS positive subscale* (score range 7–42)	62	15.2	5.1
PANSS negative subscale* (score range 7–42)	61	17.9	7.0
PANSS total score* (score range 30–210)	61	65.3	17.5
Antipsychotic medication DDD**	62	1.7	0.9
Light intensity Physical activity (min/day^−1^)	62	216	92.4
Moderate/vigorous physical activity (min/day^−1^)	62	28.7	31.0
Steps/day (steps*day^−1^)	62	5685.0	3641.0
Sedentary time (h*day^−1^)	62	8.6	1.6
VO_2peak_ (mL*kg^−1^*min^−1^), all participants	62	30.2	11.6
VO_2peak_ (mL*kg^−1^*min^−1^), participants fulfilling maximal effort criteria***	52	31.5	11.8
VO_2peak_ (mL*kg^−1^*min^−1^), participants not fulfilling maximal effort criteria****	10	33.9	7.7

Data presented in % or mean.

* PANSS, Positive and Negative Syndrome Scale;

** DDD, defined daily dose, dose equivalence estimate based on total intake of antipsychotics per day;

*** Respiratory exchange ratio≥ 1.00.

**** Respiratory exchange ratio < 1.00.

### Assessments

For further details on measurement of physical activity, sedentary time, and oxygen uptake see (24). PA and sedentary time was assessed using the ActiGraph GT3X+ (ActiGraph, LLC, Pensacola, FL, USA) worn over the left hip while awake for four consecutive days. Analyses were restricted to participants who wore the accelerometer for a minimum of 10 h per day for 2 days or more. To identify different intensities of PA, count thresholds corresponding to the energy cost of the given intensity were applied to the data set. Sedentary time was defined as all activity < 100 counts per minute (CPM), a threshold that corresponds with sitting, reclining, or lying down (25). Light intensity PA was defined as 100–2019 CPM, moderate as 2020–5998 CPM, and vigorous as CPM ≥ 5999 (26). The amount of minutes per day at different intensities was based on summing the time where the activity count met the criteria for the specific intensity. Cardiorespiratory fitness (CRF) was operationalized as VO_2peak_ measuring the highest oxygen uptake in a maximum exercise test on a treadmill (Woodway, Würzburg, Germany). Some individuals may fail to reach true VO_2max_, and for the sake of conservative reporting, we therefore use the term VO_2peak_ (mL^*^kg^−1^*min^−1^). We used a modified Balke protocol (27), where speed was held constant at 5 km·h^−1^ and the inclination angle was increased by one degree every minute until exhaustion within 6–12 min. Gas exchange was continuously sampled in a mixing chamber every 30 s by breathing into a two-way breathing valve (2700 series, Hans Rudolph Inc., Kansas City, USA). The breathing valve was connected to a Jaeger Oxycon Pro used to analyze the oxygen and carbon content. Maximal effort was assessed by the respiratory exchange ratio (RER). Participants reached the criteria when RER ≥ 1.00. Participants' psychotropic drugs prescription was presented as defined daily dose (DDD) based on approved dose recommendations. DDD provides a rough estimate of participants' drugs consumption utilizing the assumed average maintenance dose per day for each specific drug used independent of dosage form for its main indication on adults (i.e., schizophrenia for antipsychotics). For example, the DDDs for chlorpromazine and risperidone are 300 mg and 5 mg respectively. DDD were calculated in accordance with guidelines from the World Health Organization Collaborating Center for Drug Statistics Methodology (http://www.whocc.no/atcdd). Information on smoking, illness duration and medication was obtained through interview and the use of hospital records. Weight was measured without shoes in light clothing by a SECA electronic scale to the nearest 0.5 kg. Height was measured without shoes with a transportable stadiometer and set to the nearest 0.5 cm.

### Statistics

Variables that were not normally distributed (*steps/day*; *MVPA*) were log-transformed in the statistical analyses. Multiple regression analyses (all covariates entered concomitantly in the model) were employed to examine the relationships between independent variables and VO_2peak._ VO_2peak_ was used to represent the outcome, cardiorespiratory fitness. Selection of variables in the regression model was based on Pearson correlation tests between VO_2peak_, measures of physical activity and sedentary time and other variables with assumed clinical importance. Correlation coefficients are shown in Table 2. Due to high correlation between the independent variables MVPA and steps/day (*r* = 0.73) two separate regression analyses were employed using each of these two independent variables and all other selected variables. The independent variable steps/day, and not MVPA, was included in the final regression model. When testing for multicollinearity correlations between independent variables were sufficiently low (*r* < 0.70) and the variation inflation factor fell within the criteria (VIF < 10). All statistical analyses were performed using IBM SPSS (Statistical Pack- age for the Social Sciences for Windows, version 24, IBM, Inc., Chicago, IL, USA).

**Table 2:** Correlation coefficients between physical activity measures, sedentary time, and VO_2peak_.

	**Gender**	**Age**	**Light intensity physical activity**	**Moderate/vigorous physical activity**	**Steps/day**	**Sedentary time**
Light intensity physical activity	0.11	0.18	–	–	–	–
Moderate/vigorous physical activity	−0.11	−0.35**	0.09	–	–	–
Steps/day	0.16	−0.25	0.45**	0.73**	–	–
Sedentary time	−0.09	0.01	−0.44**	−0.31*	0.42**	–
VO_2peak_	−0.22	−0.64**	−0.002	0.28*	0.34**	0.02

* p < 0.05;

** p < 0.001.

## Results

As shown in Table 3, VO_2peak_ was significantly associated with gender, age and steps/day when all other covariates were controlled. Regression coefficients and standard errors can be found in the table. In a separate regression analysis encompassing MVPA in addition to the independent variables gender, age, light intensity PA and sedentary time, MVPA did not contribute to VO_2peak_. Eighty-four percent of the participants obtained the criteria for RER. The main results were unaltered in a multiple regression analysis with participants attaining RER ≥ 1.00.

**Table 3:** Regression analysis presenting explained variance in VO_2peak_.

	***R***	***R*^**2**^**	**Unstand-ardized coefficie-nt (B)**	**Standard error**	**Standar-dized coefficient (Beta)**	***t*-value**	**Variance inflation factor (VIF)**	***P*- value**
Gender	−0.164	0.027	−3.914	2.270	−0.169	−1.724	1.068	0.090
Age	−0.510	0.260	−0.481	0.090	−0.568	−5.368	1.243	< 0.001**
Light intensity physical activity	0.054	0.0008	0.008	0.015	0.067	0.569	1.535	0.572
Steps/day	0.206	0.042	11.747	5.404	0.262	2.174	1.615	0.034*
Sedentary time	0.134	0.018	0.018	0.013	0.156	1.412	1.346	0.163

N = 62 for all variables. All physical activity variables and sedentary time were assessed by accelerometer.

Partial correlation to V0_2peak_ (R), uniquely explained variance (R^2^) by each of the predictors, unstandardized coefficients (B), standard error of the coefficients, standardized coefficients (Beta), t-values, variance inflation factors (VIF), and p-values are presented in the standard regression model.

* p < 0.05;

** p < 0.001.

## Discussion

The main finding in the current study was that total daily steps was significantly associated with VO_2peak_, independently of light intensity physical activity, the amount of time spent sedentary and maximal effort during testing of oxygen consumption. Moderate-to-vigorous physical activity was not significantly associated with VO_2peak_. Our findings suggest that the amount of daily walking activity is of importance for patients with schizophrenia by contributing to the established health indicator VO_2peak_. As a measure of the total physical activity daily steps differs from the measures of physical activity at defined levels of activity. VO_2peak_ may reflect the individual's participation in physical activity of different intensity. The influence of physical activity at specific intensity levels on VO_2peak_ has been focus of previous research. A meta-regression of moderators of physical activity showed that low VO_2peak_ was associated with low levels of moderate-to-vigorous physical activity (2). In a recent study of patients with psychosis using a 6 min-walking test as a proxy for CRF, both MVPA and total PA showed significant moderate positive correlations with CRF (28). The findings in the current study indicate that repeated or prolonged sequences of walking could have favorable health effects in schizophrenia. However, the current study investigating the relations between VO_2peak_ and physical activity at different intensity levels, sedentary behavior and daily steps in patients with schizophrenia needs to be replicated.

A comparison of studies using objective assessments of PA and sedentary behavior with studies based on self-reports suggests that people with schizophrenia underestimate the amount of sedentary time and overestimate the duration of their PA (2). The objective assessment of PA is a strength of the current study. A limitation is the cross sectional design without the opportunity to draw inferences concerning causal effects. The encouragement of repeated indoor, as well as outdoor physical activity, could be organized according to personal preference and implemented as part of treatment in community-based mental health care. In conclusion, the amount of steps throughout the day contributes to VO_2peak_ in people with schizophrenia, independently of light intensity physical activity and sedentary time. Moderate-to-vigorous physical activity was not significantly associated with VO_2peak_. This may have implications for the choice of strategies when helping patients with schizophrenia improve their cardiorespiratory fitness.

## Ethics Statement

This study was carried out in accordance with the recommendations of Regional Ethics Committee of Southern and Eastern Norway (REK Sør-Øst) under file number 2014/372/REK Sør-Øst C with written informed consent from all subjects. All subjects gave written informed consent in accordance with the Declaration of Helsinki. The protocol was approved by the Regional Ethics Committee of Southern and Eastern Norway (REK Sør-Øst).

## Author Contributions

JAE and EA conceived the study. JAE, JE, EA, and JM acquired funding and approval of the ethics committee. These four researchers, in addition to EWM and OAA, contributed to study design. TLH, TTB, GB-K and JM carried out parts of the clinical testing. The manuscript has been drafted by JAE, EA, JE, OAA, GB-K, TTB, TLH, EWM, and JM. All authors read, worked on and approved the final manuscript.

### Conflict of Interest Statement

The authors declare that the research was conducted in the absence of any commercial or financial relationships that could be construed as a potential conflict of interest.
